# All in Its Proper Time: Monitoring the Emergence of a Memory Bias for Novel, Arousing-Negative Words in Individuals with High and Low Trait Anxiety

**DOI:** 10.1371/journal.pone.0098339

**Published:** 2014-06-02

**Authors:** Annuschka Salima Eden, Pienie Zwitserlood, Katharina Keuper, Markus Junghöfer, Inga Laeger, Peter Zwanzger, Christian Dobel

**Affiliations:** 1 Institute for Biomagnetism and Biosignalanalysis, University Hospital Münster, Münster, Germany; 2 Institute of Psychology, University of Münster, Münster, Germany; 3 Department of Psychiatry, University Hospital Münster, Münster, Germany; Utrecht University, Netherlands

## Abstract

The well-established memory bias for arousing-negative stimuli seems to be enhanced in high trait-anxious persons and persons suffering from anxiety disorders. We monitored the emergence and development of such a bias during and after learning, in high and low trait anxious participants. A word-learning paradigm was applied, consisting of spoken pseudowords paired either with arousing-negative or neutral pictures. Learning performance during training evidenced a short-lived advantage for arousing-negative associated words, which was not present at the end of training. Cued recall and valence ratings revealed a memory bias for pseudowords that had been paired with arousing-negative pictures, immediately after learning and two weeks later. This held even for items that were not explicitly remembered. High anxious individuals evidenced a stronger memory bias in the cued-recall test, and their ratings were also more negative overall compared to low anxious persons. Both effects were evident, even when explicit recall was controlled for. Regarding the memory bias in anxiety prone persons, explicit memory seems to play a more crucial role than implicit memory. The study stresses the need for several time points of bias measurement during the course of learning and retrieval, as well as the employment of different measures for learning success.

## Introduction

A hallmark finding in emotion research is that emotional items receive preferential processing. This has been evidenced by behavioral and neurophysiological methods for emotional scenes and faces (e.g. [1]–[3]), but also for symbolic stimuli such as words and gestures ([4]–[8], for review see [9], [10], [11]). Moreover, emotional items evoke a memory bias, with better performance for emotional than for neutral stimuli. This is independent of stimulus type, and was demonstrated for emotional pictures and scenes (e.g. [12]–[14]), for film clips (e.g. [15]), stories (e.g. [16]) and again, also for words (e.g. [17], [6], [10]).

Interestingly, this bias in the processing of, and memory for, emotional (in particular, arousing-negative) stimuli is more strongly expressed in persons suffering from anxiety disorders (e.g. [18]–[21]) or in persons with a subclinical anxious personality [22]–[25]. People with high levels of trait anxiety do not necessarily meet the diagnostic criteria for an anxiety disorder, but are particularly prone to develop one (e.g. [23], [26], [25], [27]). It is generally assumed that the differences in processing and learning of emotional stimuli, in combination with environmental and genetic predispositions, constitute the basis for the development of anxiety disorders (e.g. [28]). Thus, to understand these disorders and to develop effective psychotherapeutic treatments, it is essential to understand how emotionally arousing and negative stimuli and situations are processed and learned.

This is why the current study investigates the emergence of memory bias for stimuli with a recently acquired arousing-negative connotation (called “arousing-negative words” in the following) in high and low trait anxious persons. Learning involved the pairing of novel, word-like stimuli (pseudowords such as “muxo” or “binu”) with arousing-negative or neutral pictures. The development of the bias is monitored during learning, immediately after learning and in a follow-up two weeks later with several measures for memory performance.

How is a memory bias for emotional items best assessed? Most studies investigate this bias immediately after a learning phase, and only few studies focus on the consolidation phase after learning. During consolidation, initial memory traces are stabilized, and newly acquired memory traces are integrated into existing cortical and subcortical memory networks ([29]–[32]). Experimental studies, both with animals and humans, provide evidence that time and sleep play important roles in memory consolidation (e.g. [33]–[42]). Experimental evidence on differential consolidation for emotional and neutral word-stimuli comes from Sharot and Phelps [43] who assessed the recognition of existing words immediately and 24 hours after peripheral, unattended presentation. While recognition for neutral words deteriorated over time, performance for negative words remained stable. Hu, Stylos-Allan and Walker [44] stressed the importance of sleep for the superior consolidation of emotional over neutral items.

When measuring memory performance, a major distinction is drawn between explicit and implicit memory. Explicit or declarative memory is knowledge that can be consciously accessed, involving personal and world knowledge, such as trying to recall someone's name. In experimental learning contexts, it is most often measured by asking the participant to freely recall or recognize items from an earlier learned list. In contrast, implicit or non-declarative memory refers to memory that cannot be consciously accessed. Implicit memory is often measured indirectly, for example by word-fragment completion [45]. It has often been argued that implicit and explicit knowledge are distinct from each other, going along with distinct biological correlates [46]–[50]. On the whole, a memory bias for emotional stimuli was shown more often with explicit than implicit memory measures. Evidence for the existence of an explicit memory bias for emotional stimuli in persons with high trait anxiety comes from Eysenck and Byrne [45], Russo and colleagues [25], as well as from a meta-analytic review by Mitte [26], who analyzed data from implicit and explicit tests separately. Mitte concluded that anxiety had an impact on some explicit measures such as free recall, but not on other explicit measures such as recognition. Evidence for the existence of an implicit memory bias is thus less clear. It has been suggested in persons with high levels of anxiety by Eysenck and Byrne [45], Williams, Mathews and MacLeod [51] as well as by Williams, Watts, MacLeod and Mathews [52], [53], but could not be replicated by others [23], [54]. Given such heterogeneous findings, it remains unclear under which circumstances an implicit memory bias may exist.

Taken together, there is evidence for the existence of a memory bias on the explicit recall of emotional stimulus material (in particular for individuals high in (subclinical) anxiety), while evidence for an implicit bias is less clear. Despite the large body of literature on the topic, we are confident that we can add to this field by addressing the following issues. First, we measure (the emergence of) bias for neutral stimuli that gain their emotional connotation through learning. We thus avoid stimuli that already possess a particular valence (e.g. the word “shark”, or a picture of a shark) that was acquired during the participants' lifetime, or may even be innate. It is obviously impossible to have control over the emotional content of such stimuli. By using initially neutral stimuli – meaningless pseudowords - we aim at a better control of learning histories, learning strategies and depth of encoding. Second, we perform follow-up assessments with memory- and valence-related measures, to target consolidation processes. Until now, recognition or recall performance was most often measured immediately after the relevant task, although there is strong evidence for memory-enhancing effects at later post-training stages (cf. [55]). Third, we believe that an on-line measurement already during learning can considerably add to our understanding of the temporal characteristics of biased memories. To learn more about the development of the memory biases it is, in our view, necessary to meet these concerns.

We thus investigated memory biases in a situation where the negative valence of stimuli was acquired under controlled conditions. Using an associative statistical learning paradigm [56], [57], neutral pseudoword stimuli were associated with pictures denoting either arousing-negative or neutral concepts, to observe the emergence of potential biases during the time course of learning. Different from other applications, learning was done in a single session, not on consecutive days. The statistical word-learning paradigm is characterized by an increased conjoint probability of two events (“correct” pairings) over the time course of learning, compared to two events with random contingency (“incorrect” pairings). The learner extracts relevant cues from the information stream, without receiving feedback and usually without conscious awareness of the underlying learning principle. By repetitively presenting combinations of critical stimuli, long-term learning becomes possible. Many repetitions lead to stronger associations between stimuli, resulting in a typical learning curve. The neural basis most likely consists of Hebbian cell assemblies, which become connected to sustain language processing [58].

The associative statistical word-learning paradigm is taken as a model for language-acquisition in children and adults (cf. [59], [60]), and offers some ecological validity. The paradigm is similar to Evaluative Conditioning (EC) but differs in some aspects. EC is a form of classical conditioning where the (dis)liking of a once neutral stimulus is acquired through associative transfer of valence from a paired (dis)liked stimulus [61]. An exemplary EC-paradigm could be as follows: a neutral stimulus such as a pseudoword (conditioned stimulus; CS) is repeatedly paired with a liked or disliked, positive or negative arousing picture (unconditioned stimulus; US). As a consequence, the previously neutral pseudoword shifts in valence towards the valence of the picture it was presented with [62]. In contrast to the statistical word learning that was applied in this study, the CS in EC-paradigms is paired with more than one emotionally arousing US, which makes EC less suitable for study designs aiming at a one-to-one mapping between concept (and specific valence) and word form. Further differences are that EC is shown to be completely independent of contingency awareness of the CS-US pairing (e.g. [63]), resistant to extinction and long lasting even up to a two months follow-up [64]. Statistical learning, similar to evaluative conditioning, does not rely on – but also does not exclude - the learner's awareness of the presented associations, and can be regarded as a form of associative learning [63], [65]–[68].

Equal groups of participants, either high or low in trait-anxiety, were tested. To measure potentially biased memories, we applied a cued-recall test and a valence rating. The recall test, a translation task, explicitly tested the newly acquired meaning of the pseudowords. The valence ratings entailed a spontaneous evaluation of the pseudowords' valence, used to assess the transfer of valence from the emotionally arousing pictures to the originally neutral pseudowords. This rating was considered to tap into implicit memory for the pseudowords' meaning, especially when explicitly remembered items were removed from the data. The valence feature is considered to be part of its semantic representation (e.g. [8]) and therefore marks a step in the acquisition of the words' meaning. Note that others have also used valence ratings to investigate implicit memory processes after only few learning instances (e.g. [69]).

Given the current literature, we expected enhanced memory effects for aversive material during learning, directly after the training, and a potentiation of this bias after an additional time delay that allows for consolidation. We expected more pronounced effects in persons with high levels of anxiety, particularly in the explicit measurements. The tests were carried out before, directly after and two weeks after the training. If consolidation has a differential impact on the bias development in high and low-anxiety groups, differences between the two groups should increase with time.

## Methods

### Ethics statement

All procedures were cleared by the ethical review board of the Ärztekammer Westfalen-Lippe and subjects gave informed consent to participate. All clinical investigation has been conducted according to the principles expressed in the Declaration of Helsinki.

### Participants

The Spielberger State Trait Anxiety Inventory [70] was completed via online survey by 310 non-clinical participants. On the basis of individual scores, twenty-seven participants, scoring thirty or below in the trait-anxiety inventory (range: 20–80) were assigned to the low-anxiety group (mean trait score = 26.30, SD = 2.39; mean age 24.48, SD = 5.56). Another twenty-seven subjects scoring fifty or above were assigned to the high-anxiety group (mean trait score =  57.59, SD = 4.76; mean age 25.41, SD = 5.69). Both groups consisted of six males and twenty-one females and were matched for age and years of schooling. All participants were native speakers of German, right-handed (as assessed by the Edinburgh Handedness Inventory (Oldfield, 1971)) and did not exhibit current axis I disorders as diagnosed by the Mini-International Neuropsychiatric Interview (M.I.N.I.) [71].

### Materials

Sixty pseudowords (e.g., “binu”, “muxo”, “alep”) served as key materials and were presented auditorily. (The stimulus material and the result files are available from the corresponding author). These pseudowords were legal consonant-vowel combinations of German, taken from Breitenstein and Knecht [56], who tested the stimuli for emotional neutrality and low similarity to existing German words. The material was recorded and pre-edited with use of audacity 1.2.6. software. Recorded stimuli were edited, cut out and converted into single wave files with PRAAT software package. The average duration of the pseudowords was 773 ms (Min. = 578 ms, Max. = 894 ms, SD = 68 ms) The selected sixty pseudowords were randomly assigned to sixty pictures depicting concrete objects. Half displayed neutral objects such as a bucket or a fence, and the other half showed arousing-negative objects such as a gun or a wound. Pictures were color photos taken from Hemera software, Wikipedia Commons (http://commons.wikimedia.org) and from the International Affective Picture System [72]. Some pictures were cropped to ensure that only one object was visible and positioned in the centre.

A pre-test was carried out to ensure neutral or arousing-negative appraisal of the pictures. Thirty participants (psychology students from the University of Münster) were presented with 100 pictures (50 subjectively judged to be negative and arousing, 50 judged neutral, non-arousing). Subjects rated valence and arousal of all pictures via Self-Assessment Manikin (SAM)-scales [73]. Scaling ranged from one (very pleasant or low arousal) to nine (very unpleasant or high arousal). The thirty most negatively rated pictures differed significantly from the thirty most neutrally rated pictures (valence: *t* (49) = 15.127, *p*<.001; arousal: *t* (49) = 16.176, *p*<.001). Accordingly, these thirty arousing-negative pictures were rated more negative and more arousing (valence: MEAN = 6.84, SD = 0.88; arousal: MEAN = 6.59, SD = 0.66) than the final thirty neutral non-arousing pictures (valence: MEAN = 4.53, SD = .92; arousal: 4.35, SD = 0.81). These sixty pictures served as materials in the main experiment. According to the German version of CELEX-Database [74], the word frequency of names for the depicted objects did not differ between arousing-negative and neutral concepts, *t (*49) = −.366, *p* = .716. Note that participants who performed the pre-test rating did not take part in the main experiment.

### Procedure

During learning (called training in the following), the subject's task was to decide intuitively by button-press whether a spoken pseudoword and a visually presented object (color picture) matched, and were invited to guess. Participants were not informed about the upcoming recall and valence tests and received no feedback on their responses during training. The training consisted of five learning passes. During each learning pass, participants were confronted with one matching “correct” and one mismatching “incorrect” pseudoword-picture pair, separated by at least one other pair. Hence, at the end of learning pass five, participants had heard each pseudoword ten times, five times paired correctly (the same pseudoword-object combination) and five times paired incorrectly (the pseudoword paired with five different other objects that were pseudo-randomly chosen). Note that all pseudowords used in “incorrect” pairing were “correctly” paired with other pictures. Thus, all presented pseudowords could be associated with meaning, and all pseudowords and pictures appeared equally often. There were 120 pseudoword-picture pairs per learning pass (thirty correct arousing-negative, thirty correct neutral, thirty incorrect arousing-negative, thirty incorrect neutral), and a total of 600 pairs per training. A single learning pass lasted approximately nine minutes. The entire training lasted about fifty minutes with a pause of five minutes after the first half of the third learning pass. All trials began with a fixation cross, positioned in the centre of the screen (500 ms) Next, a pseudoword was presented via loudspeakers (≈700 ms). Another fixation cross (300 ms) and a picture (1000 ms) followed. A red exclamation mark (2500 ms) ended the trial, providing sufficient time for the subjects to decide whether pseudoword and pictured object matched. If no answer was given, the trial ended after this 2500 ms interval, and the next trial was initiated. If a button was pressed within the 2500 ms interval, the next trial began immediately.

The explicit outcome of the training was assessed via cued recall. Subjects were presented with the pseudowords in written format (cues) and were asked to write down the corresponding German word (comparable with a translation or vocabulary test). A pseudoword-valence rating served as an assessment of the transfer of valence from objects to pseudowords, and served as a measurement of access to the concept's valence by means of the paired pseudoword. Subjects were asked to spontaneously and intuitively rate the pseudowords in terms of valence. Scaling ranged from minus five (very negative) to five (very positive), with zero marked as neutral.

### Design and data analyses

Participants were tested before, immediately after, and two weeks after training. Before training, subjects completed the first valence rating of all 60 pseudowords, immediately followed by the training. After training, the second valence rating and the first cued-recall (translation) test were administered. Two weeks later, participants were confronted with the third valence rating and the second cued-recall test. This second session was carried out online (http://www.limesurvey.org/). All assessments on the first day took place in the Institute for Biomagnetism and Biosignalanalysis, affiliated to the Faculty of Medicine (Münster University). On day one, participants received written instructions but were not informed about the fact that their memory for the pseudowords would be tested.

Performance during the training, valence rating of pseudowords and responses of the cued-recall test were analyzed by mixed design ANOVAs with repeated measures. In all three analyses, the transfer of valence of the correctly paired object to the pseudoword, labeled *pseudoword affect* (arousing-negatively linked, neutrally linked) served as a within factor, and *trait anxiety* (high vs. low) served as a between factor. There was an additional within-factor in each ANOVA, varying between tests, which will be described with each analysis.

#### During training

For analysis of performance during-training, the sensitivity index d' was calculated from hits, correct rejections, misses and false alarms [75]. The additional variable in this analysis was learning pass (1–5). A 2 (*pseudoword affect*) × 5 (*learning pass*) × 2 (*trait anxiety*) design was employed. The ANOVA and post-hoc t-tests tested the effectiveness of the training, effects of valence, and potential group differences during the learning of the pseudowords (acquisition bias).

#### Cued Recall Test

Answers in the cued-recall test (translation of pseudowords into German) were treated as correct if they described the intended object (e.g., sofa), were synonyms (e.g., couch), or subordinate-category responses that were correct descriptions of the depicted object (e.g., chesterfield). Responses were regarded incorrect if they described the superordinate category (e.g., furniture), semantically related objects (e.g., armchair) or unrelated objects (e.g., scissors). Incorrect answers and misses (no answer given) were excluded from further analyses. The mean of correctly translated pseudowords was subjected to an ANOVA with the factor *session* as an additional factor, with two levels (immediately after, two weeks after). This resulted in a 2 (*pseudoword affect*) × 2 (*session*) × 2 (*trait anxiety*) mixed within/between design.

#### Valence Rating

For analysis of the pseudoword valence ratings, the additional factor was *session,* with three levels (before training, immediately after, and two weeks after training). Mean valence ratings were calculated for arousing-negatively and neutrally linked pseudowords. With a 2 (*pseudoword affect*) × 3 (*session*) × 2 (*trait anxiety*) mixed within/between design, the development of valence ratings was investigated over time, for arousing-negatively and neutrally linked pseudowords in both anxiety groups.

To assess implicit effects of pseudoword affect, for those pseudowords that were not explicitly remembered, an additional repeated-measures ANOVA with the same design was performed on the valence data, after removing all explicitly remembered stimuli for each participant individually. Since the first valence rating took place before training (thus, before any explicit learning), all sixty items (30 paired arousing-negative, 30 paired neutral) entered the analysis at this time point in all cases. For the second (directly after training) and third (two weeks after training) session, the following number of items entered the analysis: High trait anxious persons, directly after training, arousing-negative words: MEAN = 23.8, SD = 5.0, range = 11–30; neutral words: MEAN = 24.81, SD = 4.7, range = 13–30; two weeks after training, arousing-negative words: MEAN = 26.9, SD = 2.8, range = 19–30; neutral words: MEAN = 28.7, SD = 1.9, range = 21–30. Low trait anxious persons, directly after training, arousing-negative words: MEAN = 23.8, SD = 4.4, range = 16–30; neutral words: MEAN = 24.1, SD = 4.0, range = 15–30; two weeks after training, arousing-negative words: MEAN = 28.3, SD = 2.0, range = 24–30; neutral words: MEAN = 28.5, SD = 1.2, range = 26–30.

## Results

### Performance during learning

**Figure 1:**
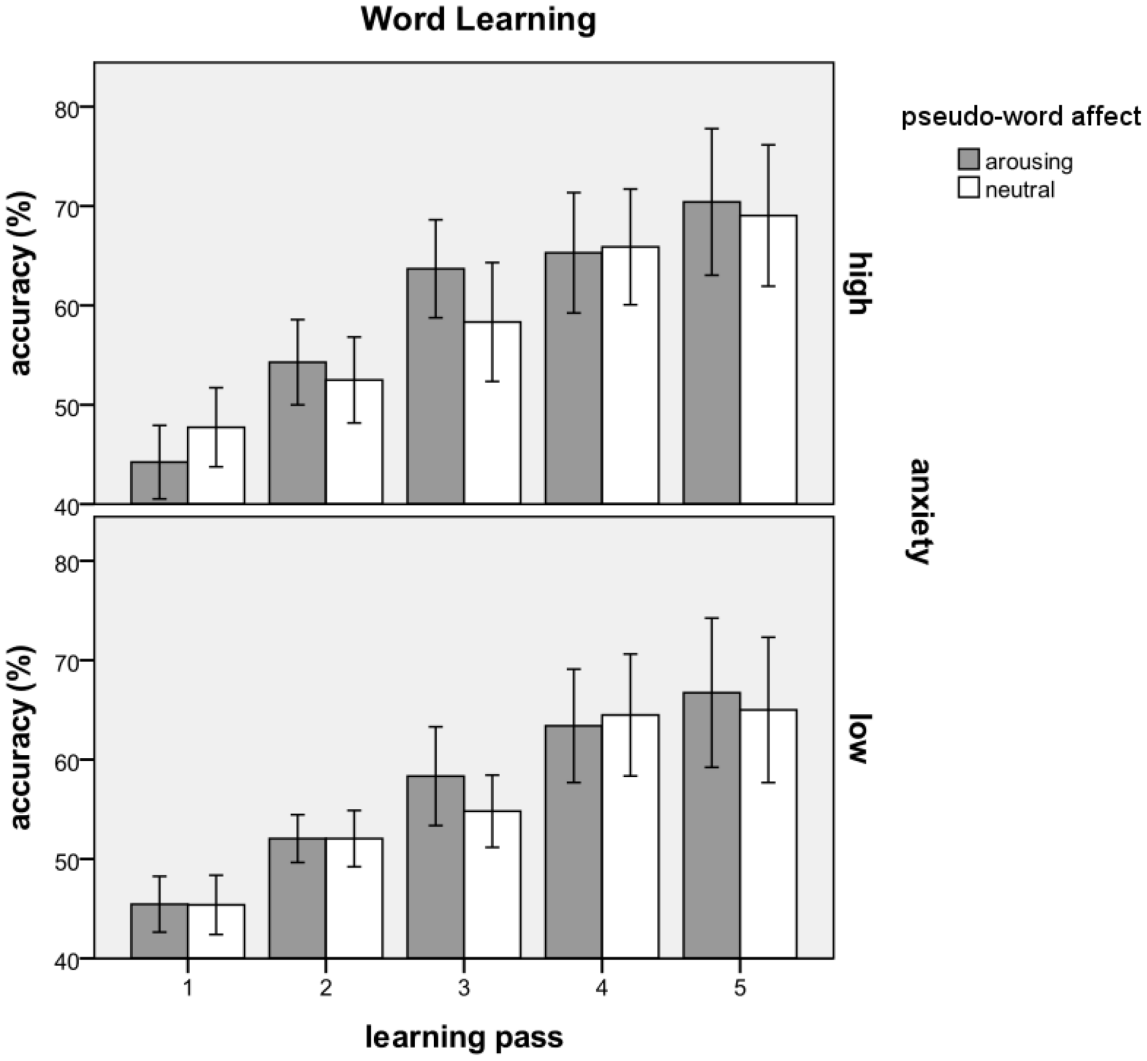
Accuracy (hits and correct rejections) during the word-learning training. The ability to differentiate between correct and incorrect pseudoword-picture pairs increased as a linear trend from a guessing rate of about fifty percent (beginning of training) to about seventy percent (end of training). Shown are averaged responses for arousing-negative and neutral pseudowords for high-anxiety subjects (upper row) and low-anxiety subjects (lower row). Pseudowords with a to-be-learned negative connotation are shown in grey; neutrally linked pseudowords are presented in white. Error bars represent one standard error.

During training, accuracy rates (hits and correct rejections) continuously increased, while false alarms and misses continuously decreased (see figure 1). The ability to differentiate between correct and incorrect stimulus pairs increased from an average guessing rate of about fifty percent (beginning of training) to about seventy percent (end of training). This was confirmed by statistical analyses (note that the statistical calculations were done on d’ values, while the figure shows accuracy rates in percent). There was a significant main effect for *learning pass*, *F* (4, 208) = 80.477; *p*<.001, best described as a linear *F* (1, 52) = 106.024; *p*<.001 and a quadratic trend *F* (1, 52) = 4.975; *p*<.030, which provided evidence for the general effectiveness of the training. However, there were no main effects for *pseudoword affect* or *trait anxiety*. Moreover, there were no significant interaction effects, except for *pseudoword affect* by *learning pass*, *F* (4, 208) = 3.875; *p* = .005. There was no difference between arousing-negative and neutral affect on learning passes 1, *t* (53) = 1.098; *p* = .277 and learning pass 5, *t* (53) = 1.405; *p* = .166 There were insignificant trends in learning pass 2, *t* (53) = 1.701; *p* = .095, and 4, *t* (53) = −1.847; *p* = .070. A significant difference emerged only in learning pass 3, *t* (53) = 2.285; *p* = .026, with better learning for arousing-negatively linked than for neutrally linked pseudowords. In all, there was little evidence for an immediate overall acquisition bias for arousing-negatively linked pseudowords, and no immediate acquisition differences between high- and low-anxiety subjects.

### Cued Recall

**Figure 2:**
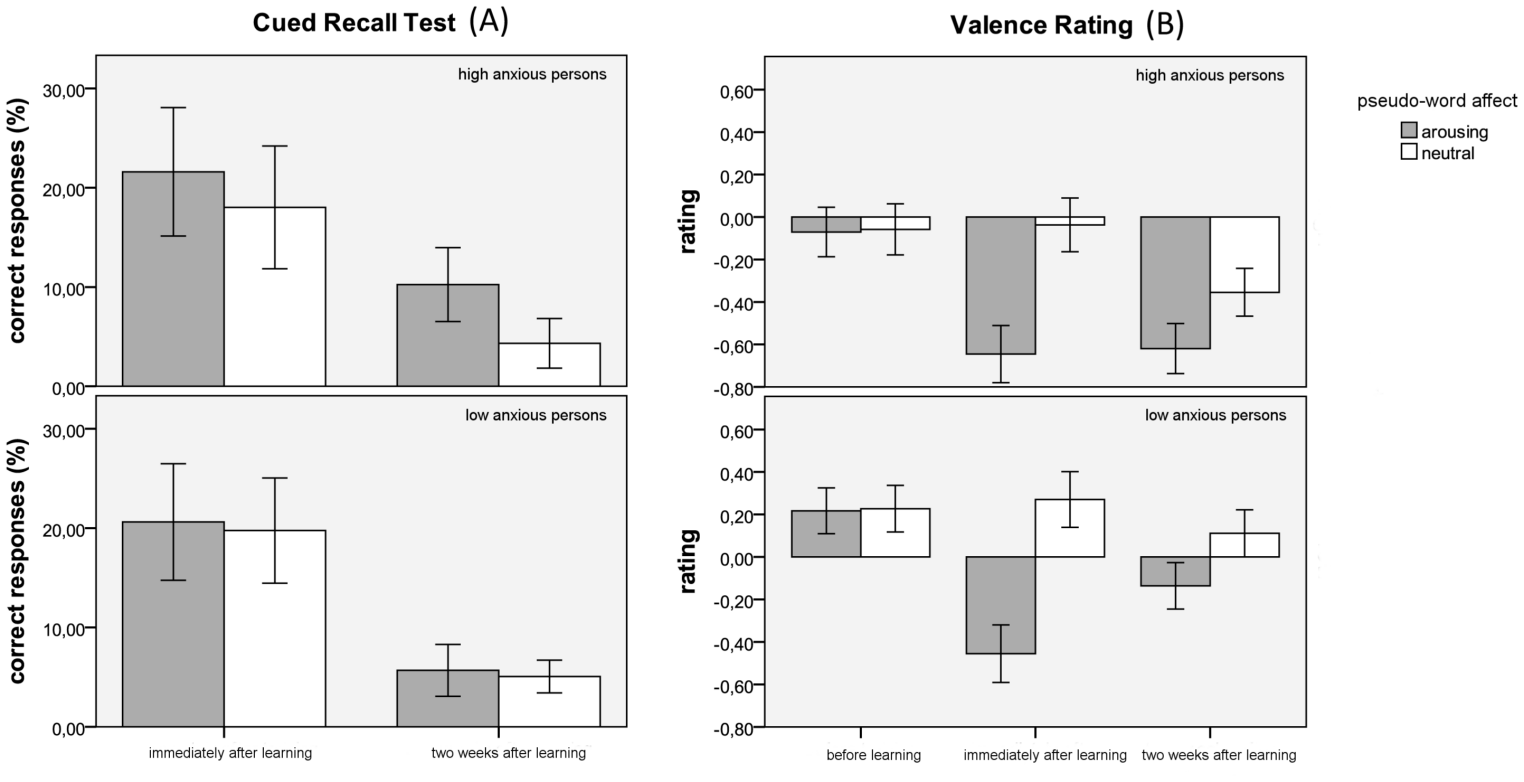
Percentage of correct responses in the cued recall test (A) and valence ratings of pseudowords (B) displayed for sessions and both high-anxiety (upper row) and low-anxiety subjects (lower row). Pseudowords with to-be-learned arousing-negative connotation are shown in grey; neutrally linked pseudowords are presented in white. Error bars represents one standard error.

Figure 2A displays the recall rates (correct translation) immediately after learning and two weeks later, for both participant groups and pseudoword types. Note that performance is displayed in percentage correct, while statistical analyses were done on absolute values. Overall, arousing-negatively linked pseudowords were recalled significantly more often than neutrally linked ones, which is reflected in a main effect for *pseudoword affect*, *F* (1, 52) = 8.787; *p* = .005. Also, recall rates were higher immediately after training than two weeks after training, as evident from the main effect of *session F* (1, 52) = 89.760; *p*<.001. Moreover, the predicted interaction between *pseudoword affect* and *trait anxiety* also reached significance *F* (1, 52) = 4.687; *p* = .035. As hypothesized, high-anxiety subjects recalled significantly more arousing-negatively linked pseudowords than neutrally linked ones *t* (26) = 3.123; *p* = .004. This explicit memory bias is not seen in the low-anxiety group *t* (26) = 0.700; *p* = .490. No other main effects or interactions reached significance.

### Pseudoword Valence Rating

Figure 2B displays the mean valence ratings separately for participant groups and pseudoword affects in three sessions. The ANOVA with *session* (before, immediately after and two weeks after training), *pseudoword affect*, and *trait anxiety* showed a main effect of *pseudoword affect*, *F* (1, 52) = 34.534; *p*<.001. Overall, subjects rated arousing-negatively associated pseudowords more negative than neutrally linked pseudowords. The rating behavior changed significantly over time, indicated by a main effect for factor *session F* (2, 104) = 11.030; *p*<.001, best described as a linear trend, *F* (1, 52) = 18.920; *p*<.001. The interaction between these two factors was also significant *F* (2, 104) = 28.697; *p*<.001. Before training, participants did not differentiate between pseudoword affects (arousing-negatively versus neutrally linked pseudowords), *t (*53) = 0.206; *p* = 0.837, confirming that pseudowords were equally neutral prior to learning. However, immediately after learning *t* (53) = 7.071; *p*<.001, and two weeks after learning *t* (53) = 4.024; *p*<.001, participants rated arousing-negatively linked pseudowords significantly more negative than neutrally linked pseudowords. There was a main effect for *trait anxiety F* (1, 52) = 7.153; *p* = .010, indicating that subjects with high levels of anxiety gave generally more negative ratings than low-anxiety persons. The group ratings were contrasted to a neutral value. Prior to training, low anxious participants rated the pseudowords as more positive than this baseline (MEAN = .222; SD = .428; *t* (26) = 2.696; *p* = .012). Immediately after learning (MEAN = −.092; SD = .544; *t* (26) =  −.884; *p* = .385) and two weeks later (MEAN = −.012; SD = .470; *t* (26) =  −.136; *p* = .893) ratings did not differ from baseline. Before the training, ratings of the high anxious persons did not differ from baseline (MEAN = −.064; SD = .667; *t* (26) =  −.501; *p* = .621). Immediately after learning (MEAN = −.341; SD = .739; *t* (26) =  −2.400; *p* = .024) and two weeks later (MEAN = −.487; SD = .485; *t* (26) =  −5.219; *p*<.001), ratings were negative and differed significantly from baseline.

The interactions *trait anxiety* × *session*, *trait anxiety* × *pseudoword affect* and *trait anxiety* × *session* × *pseudoword affect* did not reach significance. Importantly, the analysis of items that were not explicitly remembered showed identical results. All significant main effects and interactions remained (*session*, *F* (2,52) = 10.740; *p*<.001; *pseudoword affect*, *F* (1,52) = 6.985; *p* = .011; *anxiety*, *F* (1,52) = 6.402; *p* = .014; *session* by *pseudoword affect*, *F* (2, 104) = 4.012; *p* = .021). No other interaction reached significance. (Note: An ANOVA on the same valence data where ratings of the first session were subtracted from ratings given at the second and third session (baseline correction) yielded qualitatively the same results).

## Discussion

We monitored the development of a memory bias for arousing-negative pseudowords during and after learning in high and low trait-anxious individuals. The results demonstrate that no more than five learning instances in an associative statistical word-learning paradigm result in a memory bias for arousing-negatively linked pseudowords, in comparison to neutral pseudowords. This bias became evident in a cued-recall test immediately after training, as well as two weeks later. Valence ratings were more negative for arousing-negatively paired pseudowords at both time points. The bias was even present for stimuli that could not be explicitly remembered. Persons with high levels of anxiety displayed a strong memory bias in the cued-recall test. Moreover, they generally rated all pseudowords as more negative than low-anxiety persons. We discuss each of these aspects in turn.

As expected, only five correct and five incorrect pairings in an associative learning paradigm resulted in a memory bias for pseudowords that were paired with arousing-negative content. This once more evidences the high effectiveness of this paradigm for word learning, and its long-lasting effects [59], [60]. Counter to our expectation, the bias did not continuously increase during learning, but was in a transient manner only present in the third of five learning passes. In learning pass two (the first repetition of the to-be-learned material) there was only a trend that did not reach significance. At the end of the training the bias had vanished completely. It is tempting to conclude that the bias was present when contingency awareness between novel pseudowords and corresponding concepts was not (yet) present. Research on implicit learning showed that insight into underlying rules often appears suddenly, after participants already adapted to the contingencies, that is, after the emergence of implicit memory effects [76]. However, because there is no guarantee that the contingencies were implicit, either initially or throughout the learning session, the conclusion that an implicit memory bias is present only during rather initial stages of learning (i.e. the second repetition of stimulus material) should be treated with caution.

Immediately after learning and two-weeks later, a memory bias became apparent in the cued-recall task. Participants were able to “translate” more learned pseudowords to German when these had been paired with negative concepts. This nicely replicates earlier findings for an explicit memory bias for emotional stimuli (e.g. [26]), but as shown here after a very brief learning history. Though the tasks during learning and for the cued recall differ in several aspects, it is still surprising that one exhibits a clear memory bias (“translate”) and the other does not (“do they belong together?”). According to an influential model [77], [78], emotion effects are more likely to show up if rather coarse processing is at stake (provided by a subcortical route via the amygdalae) than with highly elaborate processing (provided by cortical structures). Given that the translation task is more demanding and more elaborate, this is a surprising result that begs an explanation. One line of reasoning goes as follows. Scott and colleagues [8] argued that valence features are part of the semantic representation of words. In line with this argument, we assume that the activation of such features is required in the translation task, where a one-to-one mapping of pseudoword to an existing German word is required. An elaborate activation of these features is not necessary in the matching task used during learning where only a rough, coarse match suffices for a correct response. This might explain why implicit effects are harder to find than explicit ones [26], at least for words.

However, the valence ratings did reveal differences between pseudowords that were paired with arousing-negative or neutral content, both immediately after learning and two weeks later. Because this bias remained even when explicitly remembered items were removed from the analysis, it rather seems to reflect implicit memory. Following our argumentation above, valence ratings must not necessarily activate elaborate associations, even when explicit recall fails. This might also explain why mere recognition of items as an explicit task failed to demonstrate a memory bias [26]. The activation of only a few associations might suffice for successful task completion.

Turning to differences as a function of trait anxiety, the memory bias was present in the high trait-anxiety group but not in the low trait-anxiety group. As for all participants, and as previously reported [54], [25], the bias differences between groups depended on the type of measurement used. In accordance with the outcome of the meta-study by Mitte [26], group differences were readily detectable in the explicit cued-recall test, where the predicted interaction emerged between anxiety group and pseudoword affect, with only the high-anxious participants recalling more negative than neutral words. This supports earlier findings for a memory bias for stimuli with emotional content in high-anxious individuals. For example, trait-anxious individuals and people with generalized anxiety disorder remembered more threat words than controls [25], [24]. Dowens and Calvo [79] report the same, but given that some of the reported threat words had not been presented previously, the authors concluded that the effect was due to a general response bias instead of a genuine memory bias. This is similar for the main effect of trait anxiety exhibited in our own valence ratings. Compared to persons with low anxiety levels, persons with high anxiety levels gave more negative ratings across all time points of measurement - even before learning. To explore this observation in more detail, we contrasted the group ratings to a neutral value, as baseline. Prior to training, low anxious participants rated the pseudowords as more positive than this baseline. Immediately after learning and two weeks later, ratings became neutral/slightly negative. High-anxious individuals showed a similar time course (reflected in a main effect) but their ratings were more negative overall. High-anxious participants started with neutral ratings before training. Their second and their final ratings however, were clearly negative. This was true even when explicitly recalled items were excluded. It thus appears that before any experience was made and with increasing experience, items are generally considered as more negative and all memories connected to the experimental context were somehow consolidated and stored as arousing-negative.

We interpret both, the more negative judgment before learning and the over-extension of negative ratings to all pseudoword stimuli as a generalization effect, which is currently considered a core feature of the anxiety pathology (e.g. [80], [81]. For a long time, only the hyper-reactivity to aversive stimuli, or to aversively conditioned stimuli (CS+, after pairing with an aversive US), was regarded as the basis of the anxiety pathology. The speculation that generalization of fear might be equally involved in the development of the disorder by now received support from quite a few studies (cf. [82]–[84], [81]). Additional support came from neurophysiological studies. For instance, receptive fields in sensory cortex become retuned into the direction of CS+ attributes (e.g., CS tone frequency). Importantly, this generalizes from neurons that retune to the CS+ to neurons that retune to stimuli resembling the CS+ ([85], for review see [86]), but there is a continuous decrease in retuning as the stimulus becomes less and less similar to the original CS+ [81], [86]. Thus, while this neurophysiological mechanism ascertains that potentially threatening stimuli are recognized and remembered even when they appear in slightly different form, the same mechanism might have negative consequences in persons at risk for anxiety disorders. To investigate such issues further, associative word learning offers itself as a promising tool, because the homo-, or heterogeneity of pseudowords and the corresponding emotional or neutral concepts are under full control by the researcher.

From a methodological viewpoint, our study stresses the importance of measurements at multiple time points before, during and after learning, as well as the employment of different methods to assess learning and memory.

Although some criticisms to earlier studies have been dealt with in the current study, we should point out some limitations, and make suggestions for future studies. First, we analyzed two extreme groups (high- and low-anxious individuals), a design that is well established and often applied in similar studies. However, It would be interesting to know whether persons with moderate trait anxiety exhibit a memory bias in our type of learning paradigm. Thus, future studies should include a group with average trait anxiety scores. Second, we carefully controlled individual learning histories by applying pseudoword stimuli in our training. However, the pictures that were used to link the formerly neutral pseudowords with aversive and neutral content might have evoked emotional associations of quite varying intensity in our participants. The pictures were neither presented in the cued recall test nor in the valence rating, so there are no data on this issue. Yet it is likely that the acquired emotionality varied for the pseudoword material. Stimuli that are not perceived equally frightening by all participants provide a general and challenging problem. Even loud tones and electric shocks, which might at first glance seem objectively similar and free from individual leaning histories, are perceived differently due to individually differing pain thresholds. Third, pseudowords, when paired incorrectly, were combined with both neutral and arousing-negative pictures, and thus with mixed valence. The pairing in the correct condition was always with the same picture, and thus with the same valence. Given that the differentiation between arousing-negatively and neutrally linked pseudowords was significant in the middle of training but disappeared towards the end, it can be speculated that the increasing mixture of valences in the incorrect condition caused a confusion that diminished the initial immediate memory bias effect. Future studies could check for this by keeping the valence of correct and incorrect pairings constant. Fourth, we cannot be sure that our participants did not apply an explicit memory strategy during word learning. If this is the case, the word learning would have been rather explicit and the learning of the pseudo words happened via translation of the pictures to German words. However, because we controlled for the word frequency of German words, this does not explain the observed differences between emotional and neutral words. We also performed the analyses concerning valence by excluding the explicitly remembered words. Because results did qualitatively not differ, we strongly assume that the observed effects are not due to a retrieval of corresponding German word forms. In fact we chose a short and relatively shallow learning history in order to perform these analyses. The brevity was intended to keep the participants from in-depth learning of the stimulus material. Such deep learning took place in our earlier studies [57], [59], [60], [87] and prevented an analysis of implicitly remembered items. A further issue that should be addressed in future studies concerns the consolidation interval that follows the training. We used a two-week interval to ensure that the learned material would be thoroughly consolidated. At this time point, we observed a generalization bias that did not reach statistical significance but is clearly visible in the data (see fig. 2B). To analyze the development and potential significance of this generalization bias, future studies should apply a shorter interval, or introduce additional time points of measurement that lie in between. And, as a final matter, we suggest that future studies add positive arousing stimuli and a further condition to the paradigm. Based on the literature we followed a strict hypothesis driven approach and thus applied only negative arousing and neutral stimuli. However, including a condition with positive words could clarify if persons with high levels of anxiety perform as controls for such material or worse.

In sum, our data demonstrates that only few learning instances evoke a memory bias for pseudowords paired with arousing-negative meaning. This bias is evident in explicit and implicit tests of memory, and is more strongly expressed in persons with high levels of anxiety. The learning paradigm with its associations between emotional content and neutral linguistic stimuli goes beyond classical, operant and evaluative conditioning paradigms mainly used so far in emotion research. It allows monitoring the development of a memory bias during learning, and how it evolves after learning. As such, we consider the paradigm highly suitable for the investigation of affective disorders and how they come into existence.
